# Angioedema in the emergency department: a practical guide to differential diagnosis and management

**DOI:** 10.1186/s12245-017-0141-z

**Published:** 2017-04-13

**Authors:** Jonathan A. Bernstein, Paolo Cremonesi, Thomas K. Hoffmann, John Hollingsworth

**Affiliations:** 1 Division of Immunology/Allergy, University of Cincinnati, 231 Albert Sabin Way, Cincinnati, OH 45267 USA; 2 Department of Emergency Medicine, E. O. Galliera Hospital, Genoa, Italy; 3 Department of Oto-Rhino-Laryngology, Head and Neck Surgery, Ulm University Medical Center, Ulm, Germany; 4 Department of Emergency Medicine, University Hospital, Aintree, Liverpool, UK

**Keywords:** Angioedema, Emergency department, Histamine-mediated, Bradykinin-mediated, Guideline

## Abstract

**Background:**

Angioedema is a common presentation in the emergency department (ED). Airway angioedema can be fatal; therefore, prompt diagnosis and correct treatment are vital.

**Objective of the review:**

Based on the findings of two expert panels attended by international experts in angioedema and emergency medicine, this review aims to provide practical guidance on the diagnosis, differentiation, and management of histamine- and bradykinin-mediated angioedema in the ED.

**Review:**

The most common pathophysiology underlying angioedema is mediated by histamine; however, ED staff must be alert for the less common bradykinin-mediated forms of angioedema. Crucially, bradykinin-mediated angioedema does not respond to the same treatment as histamine-mediated angioedema. Bradykinin-mediated angioedema can result from many causes, including hereditary defects in C1 esterase inhibitor (C1-INH), side effects of angiotensin-converting enzyme inhibitors (ACEis), or acquired deficiency in C1-INH. The increased use of ACEis in recent decades has resulted in more frequent encounters with ACEi-induced angioedema in the ED; however, surveys have shown that many ED staff may not know how to recognize or manage bradykinin-mediated angioedema, and hospitals may not have specific medications or protocols in place.

**Conclusion:**

ED physicians must be aware of the different pathophysiologic pathways that lead to angioedema in order to efficiently and effectively manage these potentially fatal conditions.

## Background

**Fig. 1:**
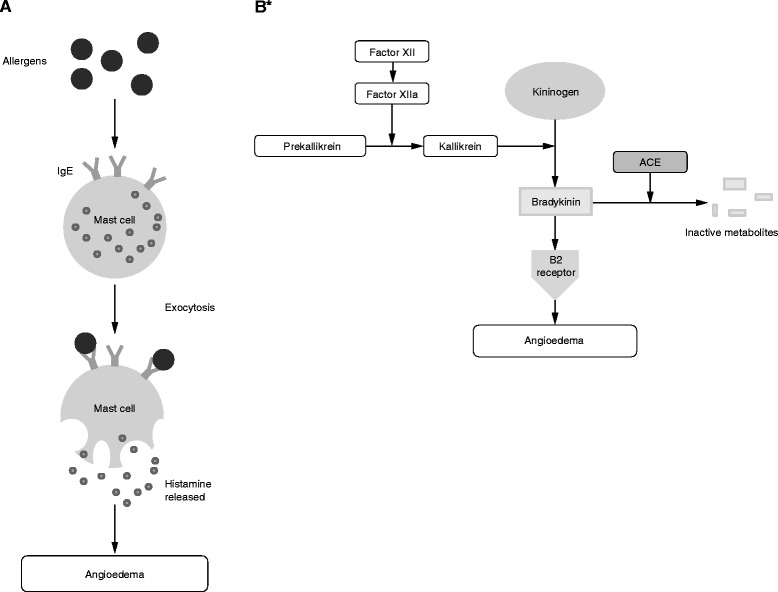
Schematic of biochemical pathways responsible for **a** histamine-mediated angioedema [62] and **b** bradykinin-mediated angioedema [26]. *C1 esterase inhibitor disrupts the action of factor XIIa and kallikrein. Ecallantide inhibits the action of kallikrein. Icatibant blocks bradykinin B2 receptors. *ACE* angiotensin-converting enzyme, *IgE* immunoglobulin E

Angioedema is a relatively common presentation in the emergency department (ED). The lifetime prevalence of angioedema and/or urticaria in the United States is about 25% and results in more than one million ED visits each year [1, 2]. Angioedema is mediated by several mechanisms, including histamine and bradykinin (Fig. 1). Diagnosis of the specific type of angioedema is essential for appropriate treatment [3]; however, many ED physicians may not know how to distinguish different types of angioedema or how to effectively treat less common presentations [4].

Each year in the USA, angioedema or allergic reactions lead to more than one million ED visits [2]. Of these, approximately 110,000 are coded as angioedema (either hereditary or acquired) compared with 979,400 coded as allergic reactions [2]. Approximately 42.5% of the visits coded as allergic reactions also include a code for urticaria [2]. Between 2280 and 5000 visits to US EDs each year are attributable to hereditary angioedema (HAE) [5, 6], accounting for a rate of 1.87:100,000 ED visits [5]; however, these figures may underrepresent the true level of angioedema-related ED use [2, 5, 6]. Similar data from Italy suggest that 0.37% of all ED visits are related to angioedema [7], and a recent Canadian study estimated that 1:1000 ED visits are angioedema related [8]. Finally, a survey in the UK revealed that 30% of patients with hereditary or acquired angioedema have visited the ED [9].

As ACEi prescriptions have become more common, the prevalence of ACEi-induced angioedema has increased substantially [10–14], accounting for 30% of all ED visits for angioedema in the USA (0.7:10,000 ED visits) and up to 60% in the West Indies [10, 11, 14].

Hospitalization can be used as a proxy measure of the severity of angioedema. Patients with undifferentiated angioedema (i.e., including both histamine- and bradykinin-mediated angioedema) visiting the ED are admitted for inpatient care (11% of ED visits) more frequently than patients with allergic reactions (2.2% of ED visits) [2, 5]. Hospitalization rates following ED visits for HAE (45–50%) and ACEi-induced angioedema (42–66%) are even higher [5, 6, 10, 12]. Hospitalizations for angioedema have increased over the last 15 years, from 3.3 to 3.4:100,000 admissions in 1998–2000 to 4.0:100,000 in 2005 and 5.4:100,000 in 2009 [15, 16]. The rise is thought to be related to increased prescribing of ACEis over this time period [15, 16].

Mortality data for angioedema are lacking; however, one study demonstrated a small but ever-present risk of death by asphyxiation in patients with HAE with development of fatal laryngeal attacks within as little as 15 min [17]. Crucially, the risk of death is three- to nine-fold higher in patients who have not received a confirmed diagnosis of HAE, emphasizing the importance of preparation and awareness in preventing adverse outcomes [17]. Although angioedema with a well-defined bradykinin-mediated pathogenesis is relatively rare, most ED staff will likely encounter a case at some point in their career. Therefore, awareness of bradykinin-mediated angioedema is important.

Because bradykinin-mediated angioedema is uncommon, there generally are not protocols in place in the ED and there is a lack of immediate access to appropriate drugs for bradykinin-mediated angioedema. For example, a recent survey of British EDs demonstrated that medications required to treat bradykinin-mediated angioedema were available in the majority of hospitals with specialist immunology services, but were not readily accessible in the ED (e.g., located in the main pharmacy). Additionally, only half the hospitals surveyed had established guidelines for the use of these medications [18].

Lack of protocols and access to medications can lead to treatment errors and poor outcomes for ED patients presenting with bradykinin-mediated angioedema [19, 20]. This paper reports the findings and recommendations of two expert panels of 16 international experts in angioedema and emergency medicine convened during 2013 [21, 22]. The aim of this paper is to provide practical guidance on the early identification of bradykinin-mediated angioedema in the ED to improve the diagnosis and outcomes of angioedema attacks.

## Review

### Angioedema: subtypes and characteristics

Angioedema is a transient subcutaneous or submucosal swelling that is non-pitting when pressure is applied [1]. Angioedema is distinct from edema, which is caused by accumulation of fluid in the interstitium and characterized by persistent pitting with pressure. Angioedema can be mediated by histamine, bradykinin, or other mechanisms [1].

### Histamine-mediated angioedema

Histamine-mediated angioedema often presents with urticaria and episodes of swelling that usually subside within 24–37 h. Histamine-mediated angioedema, also called allergic angioedema, is a type I immunoglobulin E-mediated hypersensitivity immune response of mast cell degranulation. This reaction occurs with previous sensitization to allergens such as insect stings, foods, and drugs [1].

### Nonhistamine-mediated angioedema

Bradykinin-induced angioedema is the most common cause of nonhistamine-mediated angioedema. Pseudoallergic angioedema and idiopathic angioedema originate from mechanisms that involve neither histamine nor bradykinin. These forms of angioedema are rare [23], therefore, this review will focus on histamine- and bradykinin-mediated angioedema. Bradykinin-mediated angioedema comprises three distinct types: hereditary angioedema (HAE), acquired angioedema, and angiotensin-converting enzyme inhibitor (ACEi)-induced angioedema [1].

#### Hereditary angioedema

Most cases of HAE arise from mutations in the gene encoding for C1 esterase inhibitor (C1-INH), resulting in either low plasma concentrations of C1-INH (HAE type I) or normal concentrations of functionally impaired C1-INH (HAE type II) [1]. HAE affects approximately 1:50,000 people in the general population [22]. The mechanism of a third type of HAE with normal C1-INH concentrations and function [1] is not yet fully understood [24]. Attacks in patients with HAE with normal C1-INH are similar to those in patients with HAE types I and II.

#### Acquired angioedema

Bradykinin-mediated angioedema also can develop later in life and is known as acquired angioedema. This condition is very rare, with an approximate prevalence of 1:100,000 to 1:500,000 in the general population [25]. The symptoms of acquired angioedema are the same as those for HAE; the distinguishing characteristic of acquired angioedema is that almost all cases are diagnosed during or after the fourth decade of life and are often associated with an underlying lymphoproliferative disorder and/or antibodies directed against C1-INH [25].

#### ACEi-induced angioedema

Another cause of bradykinin-mediated angioedema is associated with ACEis [26]. Angiotensin-converting enzyme is one of the two enzymes that degrade bradykinin; ACEis can cause accumulation of bradykinin that results in angioedema (ACEi-induced angioedema) [26]. Symptoms of ACEi-induced angioedema are usually localized in the face or upper aerodigestive tract; the main characteristic is erythema (without itching) lasting 24–72 h, followed by spontaneous remission [27]. Reports of rare abdominal involvement have been published [28]. ACEi-induced angioedema has been reported as a side effect affecting 0.1–0.7% of patients and up to 1.6% in some studies [29]; a large US study reported an incidence of 0.2% [30]. As the prevalence of cardiovascular conditions increases with age, ACEi-induced angioedema is likely to present more frequently in patients >40 years of age [11]. ACEi-induced angioedema has been shown to be more prevalent in female and black patients [30, 31].

#### Angioedema associated with other drugs

Less commonly, an increased risk of nonhistaminergic angioedema has been associated with other classes of drugs, including nonsteroidal anti-inflammatory drugs (NSAIDs), antibiotics, and angiotensin receptor blockers (ARBs) [7, 32]. NSAID-related angioedema is estimated to occur in 0.1–0.3% of persons exposed to NSAIDs [33, 34]. Patients with underlying diseases such as asthma and chronic urticaria are at much higher risk, and up to 35% of patients will have a reaction upon exposure to NSAIDs. There are multiple mechanisms of action for NSAID-induced angioedema, including COX-1 inhibition leading to production of the inflammatory mediators, cysteinyl leukotrienes, and IgE-mediated hypersensitivity [35]. NSAID-mediated angioedema can be managed by stopping the NSAID and treating the angioedema in a manner similar to histaminergic angioedema. For ARBs, the angioedema incidence rate per 1000 person-years was 1.66 (95% CI 1.47, 1.86) [32].

The development of asymmetric angioedema in association with recombinant (r) tissue plasminogen activator (tPA) therapy for acute ischemic stroke has become a recognized phenomenon since first reports appeared in the literature [36]. Depending on the reporting center, rates of rtPA-associated angioedema range from 1.2 – 5.1% of acute ischemic stroke patients treated with rtPA [36–40]. Additionally, concurrent use of ACEis seems to increase risk [36, 37]. Administration of rtPA not only activates components of the complement system including histamine but also leads to plasmin-mediated release of bradykinin [40, 41]. While most cases of rtPA-associated angioedema are mild and resolve over 24 h, some cases are rapidly progressive and life threatening.

### Distinguishing histamine- versus bradykinin-mediated angioedema

**Fig. 2:**
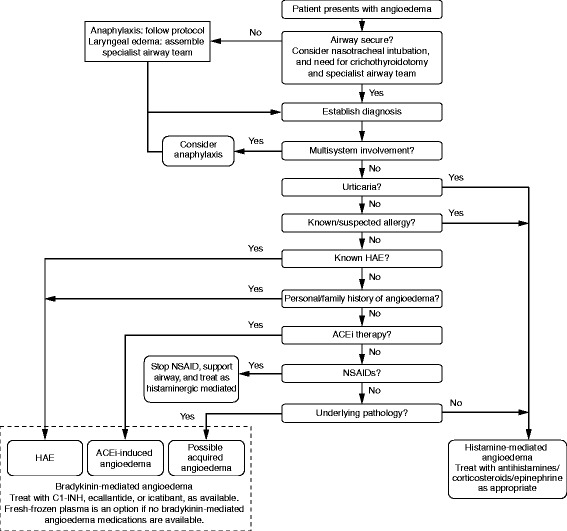
Flow diagram of diagnosis of angioedema in the emergency department [21, 42]. *ACEi* angiotensin-converting enzyme inhibitor, *HAE* hereditary angioedema

**Fig. 3:**
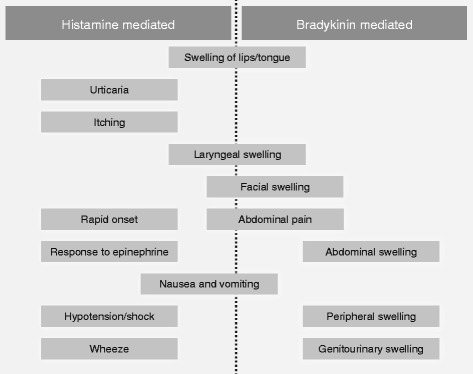
Distinguishing histamine- versus bradykinin-mediated angioedema

To ensure that patients are managed correctly, identification of the underlying cause of angioedema on presentation is essential. Unfortunately, no validated, rapid, point-of-care diagnostic test is available to differentiate a bradykinin-mediated from a histamine-mediated attack; however, a number of distinguishing features can guide the diagnosis (Fig. 2 [21, 42] and Fig. 3).

Urticaria is common in histamine-mediated angioedema. A recent Canadian study found that 29.8% of patients presenting with angioedema also had urticaria, and this was significantly associated with triggers such as insect stings, certain foods, or drugs (other than NSAIDs and ACEis) [8]. Itching is not usually associated with bradykinin-mediated attacks. Urticaria does not occur with bradykinin-mediated angioedema (hereditary or ACEi induced; Fig. 3).

**Fig. 4:**
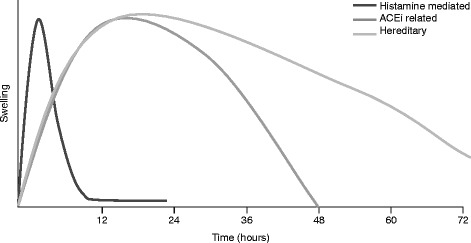
Schematic representation of angioedema attack onset and duration. Histamine-mediated angioedema attacks tend to have rapid onset and resolution. Bradykinin-mediated angioedema usually develops more slowly and can persist for ≤5 days, although angiotensin-converting enzyme inhibitor (ACEi)–induced angioedema will usually resolve ≤48 h once the drug is discontinued

Speed of onset also may be a differentiating factor (Fig. 4 [21]). Histamine-mediated angioedema can occur quickly (≤1 h of exposure to allergens). Hereditary and acquired angioedema symptoms usually have a slower, progressive onset and develop over several hours, but occasionally can develop quickly, or appear to do so (e.g., if an attack starts while a patient is sleeping).

Untreated hereditary and acquired angioedema attacks tend to be more severe and persistent than histamine-mediated angioedema attacks, typically persisting for 48–72 h and occasionally up to 5 days [43]. Also, bradykinin-mediated attacks are more likely to have abdominal involvement (Fig. 3) [21] with at least 50% of attacks involving the abdomen [44]. If a patient with a prior diagnosis of HAE presents with abdominal pain and/or swelling, gastrointestinal angioedema should be considered in the differential diagnosis, even if the patient has not previously experienced similar attacks [45]. In cases with no prior diagnosis of HAE, a differential diagnosis is much more difficult. Patient history is vital in patients presenting with abdominal pain. A history of recurrent abdominal pain and swelling, especially if accompanied by a family history of similar symptoms, may suggest HAE [46].

Response to treatment with antihistamines, corticosteroids, and epinephrine may distinguish histamine- and bradykinin-mediated angioedema. Histamine-mediated angioedema will respond to treatment with antihistamines, corticosteroids, and epinephrine, whereas bradykinin-mediated (including hereditary, acquired, and ACEi-induced) angioedema will not. Although response or lack of response to treatment is not an appropriate diagnostic measure in the ED, the effect of treatment can be a useful clinical clue for follow-up and subsequent diagnosis.

#### Confirmatory tests

A diagnosis of hereditary or acquired angioedema can be confirmed with blood tests; however, currently available blood tests cannot confirm ACEi-induced angioedema [1, 21, 47, 48] (Table 1). In a patient presenting with new-onset isolated angioedema with or without a family history, consider obtaining a screening C4 level to aid in diagnosis; 25% of HAE cases may be spontaneous mutations and therefore may not be associated with a family history. Results of blood tests taken during an attack are unlikely to be available soon enough to inform decisions in the ED, but can be useful for follow-up and future management.

**Table 1:** Results of diagnostic tests to help distinguish among angioedema types [1, 21, 47, 48]

	C1-INH concentration	C1-INH function	C4 concentration	Tryptase concentration^*^
HAE type I	Low	Low	Low	Normal
HAE type II	Normal or High	Low	Low	Normal
HAE with normal C1-INH	Normal	Normal	Normal	Normal
Acquired AE	Low	Low	Low	Normal
ACEI-induced AE	Normal	Normal	Normal	Normal
Histamine-mediated anaphylaxis	Normal	Normal	Normal	Normal or Elevated

^*^In blood drawn within 4–6 h of onset of attack

*ACEI* angiotensin converting enzyme inhibitor, *AE* angioedema, *C1-INH* C1 inhibitor, *HAE* hereditary angioedema

### Angioedema: management in the ED

**Fig. 5:**
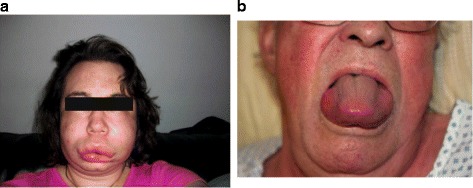
Presentation of angioedema in the emergency department. **a** Facial/lip edema (Ishoo stage I). **b** Tongue edema (Ishoo stage III). Image **a** obtained from www.haeimages.com

Presentation of angioedema in the ED will likely fit one of the four categories:

- Swelling of the face (including hemifacial swelling) or lips (Fig. 5a)

;
- Swelling of the tongue (Fig. 5b);
- Laryngeal swelling;
- Abdominal swelling, pain, or discomfort, which can be severe.

Peripheral cutaneous angioedema also is common; however, patients may not attribute the same level of risk to cutaneous angioedema as with angioedema at other sites, so presentation in the ED may be less frequent. These categories of angioedema are not mutually exclusive, and swelling of multiple sites may occur.

#### Upper airway management

Angioedema can progress rapidly. The first step is to consider whether the airway is “safe” or not. If it is not safe, and no time is available to make further assessments, the local anaphylaxis protocol should be followed and mechanical intervention may be needed, either by intubation or cricothyrotomy/tracheotomy [42]. Visualization, ideally by nasopharyngeal laryngoscopy, should be considered in patients with stridor or hoarseness to evaluate the degree of laryngeal angioedema [21]. The need for intubation is significantly greater with presentations of laryngeal or pharyngeal involvement versus swelling of the lips and face. As a general rule, if swelling is in front of the teeth, drug treatment is likely to be sufficient; if swelling is behind the teeth, mechanical airway management should be considered [49]. The incidence of intubation increases with age [50].

If intubation is required, a nasopharyngeal or endotracheal airway should be considered as first choice [21]. Bradykinin-mediated angioedema commonly affects the lips and tongue, potentially obstructing the oropharyngeal pathway, whereas the nasal passage is unlikely to be obstructed. In this case, supraglottic devices (e.g., laryngeal masks) are not appropriate. The gold standard of airway management for bradykinin-mediated angioedema is an awake nasopharyngeal intubation. Bradykinin-mediated angioedema (hereditary, acquired, ACEi induced) can be triggered by mild trauma; thus, oral and laryngeal edema can be worsened by visualization and intubation attempts. Airway management of a patient with laryngeal angioedema is fraught with danger. Before attempts at intubation, a team experienced in nasopharyngeal intubation and the emergency delivery of a surgical airway should be summoned.

Although stabilization of the airway is the highest priority, every effort should be made to determine the cause of the angioedema attack (known drug or food allergies, existing hereditary or acquired angioedema, ACEi use, family history, etc.) to ensure that the attack is managed appropriately.

### Medications for histamine-mediated angioedema

For histamine-mediated angioedema and angioedema of undifferentiated etiology, standard treatment includes H_1_ and H_2_ antagonists and oral corticosteroids [21]. Airway swelling or hypotension are indications for epinephrine at a dose of 0.2–0.5 mg administered intramuscularly [51]. In the absence of anaphylaxis, epinephrine is not indicated for nonlife-threatening symptoms that do not involve the airway.

### Medications for bradykinin-mediated angioedema

Several medications are available for the treatment of acute HAE attacks (Table 2). Although these medications are not US Food and Drug Administration approved for ACEi-induced or acquired angioedema, recent and ongoing research suggests a broader role in treatment of bradykinin-mediated attacks in patients who do not have HAE [21, 40]. C1-INH concentrates, which are derived from pooled donor plasma, are available. These C1-INH replacement products inhibit factor XII and kallikrein activity, which reduces bradykinin production [21]. Berinert® (CSL Behring, LLC, Kankakee, IL, USA) is approved in the United States for the treatment of acute abdominal, facial (includes tongue and oropharynx), or laryngeal HAE attacks in adults and children. Cinryze® (Shire, Lexington, MA, USA) is approved for the preventative treatment of HAE in adults and is used off label to treat HAE attacks. Ruconest® (Salix Pharmaceuticals, Inc., Raleigh, NC, USA) is a recombinant C1-INH product approved for the treatment of acute HAE attacks in adults and adolescents.

**Table 2:** Targeted treatment of HAE [21, 63–66]

Drug	FDA-approved indication	Mechanism of action	Dose/route/price^*^	Time to onset of symptom relief	Adverse effects
Plasma-derived C1-INH (Berinert)	Acute abdominal, facial, or laryngeal HAE attacks in adult and pediatric patients (no lower age limit established)	C1-INH protein replacement	20 units/kg IV $8991 (1500 units)	Median: 48 min	Common: dysgeusia Rare: anaphylaxis, thrombosis Theoretical: blood-borne infections
Plasma-derived C1-INH (Cinryze)	Prophylaxis of HAE attacks in adults and pediatric patients ≥11 years of age	C1-INH protein replacement	1000 units IV $5704 (1000 units)	Median: 30 min	Common: dysgeusia Rare: anaphylaxis, thrombosis Theoretical: blood-borne infections
Recombinant C1-INH (Ruconest)	Acute HAE attacks in adults and pediatric patients ≥11 years of age; effectiveness not established for laryngeal attacks	C1-INH protein replacement	50 units/kg IV $12,142 (4200 IU)	Median: 90 min	Common: sinusitis, rash, pruritus Rare: anaphylaxis
Ecallantide (Kalbitor)	Acute HAE attacks in patients ≥12 years of age	Plasma-kallikrein inhibitor	30 mg SC $14,090 (30 mg)	Median: 67 min	Common: headache, nausea, pyrexia, injection site reactions Uncommon: anaphylaxis (must be administered by a health care professional)
Icatibant (Firazyr)	Acute HAE attacks in adults ≥18 years of age	Bradykinin**-**2 receptor antagonist	30 mg SC $10,037 (30 mg)	Median: 2 h	Common: injection site reactions, pyrexia, increased transaminases, dizziness Theoretical: worsening of an ongoing ischemic event

^*^US retail pricing obtained from www.drugs.com on April 5, 2017

Kalbitor® (ecallantide, Shire., Lexington, MA, USA) is a recombinant plasma kallikrein inhibitor genetically engineered to target kallikrein-mediated bradykinin production. The product is approved for the treatment of acute HAE attacks in patients ≥12 years of age. Anaphylaxis occurred in 3% of patients during clinical trials, necessitating administration by a health care professional who can manage hypersensitivity reactions and observation for ≥1 h following administration [21]. Icatibant is a synthetic selective bradykinin-2 receptor antagonist that inhibits the vascular effects of bradykinin and is approved for treatment of HAE attacks with subcutaneous administration by either patients or physicians [21]. No head-to-head comparative trials of these agents are available. About 10% of patients may require a second dose for incomplete response or symptom recurrence [21, 40, 41, 52]. Several clinical trials have recently been reported using ecallantide or icatibant for ACEi-induced angioedema. Lewis et al. compared the addition of ecallantide to standard therapy in patients with mild to moderate ACEi-induced angioedema with an endpoint of discharge eligibility within 6 h of treatment. Discharge eligibility was similar in patients receiving ecallantide or placebo [53]. Using a shorter discharge time as an endpoint, Bernstein et al. found that eight of 26 (31%) patients receiving ecallantide versus five of 24 (21%) patients receiving placebo were discharged in ≤4 h (95% confidence interval, −14 to 34%) [52]. Bas et al. reported significantly shorter time to symptom relief with icatibant than with standard glucocorticoid and antihistamine therapy (2.0 vs 11.7 h, respectively; *p* = 0.03) [40], corroborating the results of a case series [54]. Although not US Food and Drug Administration approved for acute treatment, fresh-frozen plasma (FFP) also may be used for ACEi-induced and other bradykinin-mediated angioedema. FFP provides volume replacement and is effective in most cases of bradykinin-mediated angioedema with onset of symptom relief in approximately 30 to 90 min [55]. FFP also is less expensive than targeted treatments, but can cause hypersensitivity reactions and rarely, worsening angioedema symptoms [21, 22]. H_1_ and H_2_ antagonists, oral corticosteroids, and epinephrine are unlikely to improve bradykinin-mediated angioedema [21].

For angioedema associated with tPA, the mainstay of treatment thus far has been the combination of intravenous corticosteroids and antihistamines. Given the effectiveness of novel treatments such as icatibant and ecallantide for patients with HAE as well as ACEi-associated angioedema, these drugs also hold promise for angioedema associated with thrombolytic therapy.

#### Discharge

Patients without involvement of the tongue or larynx and with no other threat to the airway (Ishoo stage I; Ishoo grading is not validated but may be helpful as a guide) can be discharged after a number of hours of observation (≥2–6 h or longer as necessary) to ensure that symptoms have begun to resolve with no indication of development of airway obstruction. Discharge should not occur unless the airway has been assessed as Ishoo stage I or below. Patients with mild angioedema of the lips may be discharged with specific treatment if no further progression is observed [21, 49]. On discharge, if possible, patients should be prescribed suitable medication, to control recurrent symptoms with referral for follow-up [21].

For patients with histamine-mediated or undifferentiated angioedema, discharge plans should include epinephrine and follow-up with an angioedema specialist to determine the appropriate treatment [21]. Patients with HAE should have access to targeted treatment that can be administered outside of the health care setting, for example, icatibant or a C1-INH inhibitor. For patients with nonhistamine-mediated angioedema, ACEis should be discontinued immediately with prescription of an alternative class of medication with a different mechanism of action. Patients should be informed that angioedema may occur or recur several weeks after cessation of ACEi therapy [56]. For patients who had a life-threatening attack of angioedema, both ACEis and ARBs should be avoided. For patients with less severe reactions to ACEi, ARBs may be used with close monitoring in patients with conditions that may particularly benefit from renin-angiotensin-aldosterone inhibition, such as chronic heart failure [36, 39].

#### Follow-up

Patients without a prior diagnosis should be referred to their primary care physician, an immunologist, or an allergy clinic on discharge depending on the suspected cause of angioedema. If HAE is suspected, the patient should be referred to an immunologist with experience managing HAE to confirm the diagnosis and discuss possible use of prophylactic and/or on-demand therapies [21]. Results of blood tests taken in the ED during an attack, while unlikely to guide acute treatment, can be valuable for subsequent follow-up in the outpatient setting.

#### Preparing your ED

To ensure optimal treatment of patients presenting with angioedema, each ED would benefit from having an established protocol, algorithm, or management plan in place that is displayed or easily accessible. Recently published national and international guidelines may be a good basis for this plan [18, 21, 22, 42, 43, 47, 48, 57–61]. Beyond training staff to recognize, differentiate, and manage angioedema in the ED, access to effective and well-tolerated drugs to treat bradykinin-mediated angioedema is vital.

## Conclusions

Angioedema is a relatively common presentation in the ED and is potentially fatal. Angioedema management in the ED starts with assessing and securing the airway while initiating specific treatment. To ensure appropriate treatment and management, determination of whether the angioedema is mediated by histamine or bradykinin is essential. With the current lack of a reliable point-of-care test to distinguish the two pathophysiologies, ED physicians should familiarize themselves with available indicators to help guide treatment decisions. Histamine-mediated angioedema should be treated with H_1_ and H_2_ antagonists and oral corticosteroids along with epinephrine, as appropriate. Patients with HAE should receive a medication indicated for treating HAE such as a C1-INH inhibitor, ecallantide, or icatibant. Other causes of bradykinin-mediated angioedema may be treated with FFP. Hospitals should ensure that adequate procedures and treatments are in place for the management of angioedema.

## Abbreviations

ACEi: Angiotensin-converting enzyme inhibitor
ARB: Angiotensin receptor blocker
C1-INH: C1 esterase inhibitor
ED: Emergency department
FFP: Fresh-frozen plasma
HAE: Hereditary angioedema
NSAID: Nonsteroidal anti-inflammatory drug
rtPA: Recombinant tissue plasminogen activator
tPA: Tissue plasminogen activator
